# The involvement of Toll‐like receptor 9 in the pathogenesis of erosive autoimmune arthritis

**DOI:** 10.1111/jcmm.13735

**Published:** 2018-07-11

**Authors:** Anita Fischer, Shahla Abdollahi‐Roodsaz, Christina Böhm, Birgit Niederreiter, Brigitte Meyer, Anthony C. Y. Yau, Erik Lönnblom, Leo A. B. Joosten, Marije Koenders, Christian H. K. Lehmann, Diana Dudziak, Gerhard Krönke, Rikard Holmdahl, Günter Steiner

**Affiliations:** ^1^ Division of Rheumatology Internal Medicine III Medical University of Vienna Vienna Austria; ^2^ Department of Rheumatology Radboud University Nijmegen Medical Centre Nijmegen The Netherlands; ^3^ Division of Rheumatology Department of Medicine New York University School of Medicine New York NY USA; ^4^ Medical Inflammation Research Department of Medical Biochemistry and Biophysics Karolinska Institute Stockholm Sweden; ^5^ Department of Dermatology University Hospital Erlangen Friedrich‐Alexander University of Erlangen‐Nürnberg Erlangen Germany; ^6^ Department of Internal Medicine 3 ‐ Rheumatology and Immunology Friedrich‐Alexander‐University Erlangen‐Nürnberg (FAU) Erlangen Germany; ^7^ Ludwig Boltzmann Cluster for Arthritis and Rehabilitation Vienna Austria

**Keywords:** arthritis, bone erosion, inflammation, osteoclastogenesis, toll‐like receptor

## Abstract

Endogenous nucleic acids and their receptors may be involved in the initiation of systemic autoimmune diseases including rheumatoid arthritis (RA). As the role of the DNA sensing Toll‐like receptor (TLR) 9 in RA is unclear, we aimed to investigate its involvement in the pathogenesis of autoimmune arthritis using three different experimental models of RA. The data obtained revealed involvement of TLR9 in the T cell‐dependent phase of inflammatory arthritis. In rats with pristane‐induced arthritis (PIA), TLR9 inhibition before disease onset reduced arthritis significantly and almost completely abolished bone erosion. Accordingly, serum levels of IL‐6, α‐1‐acid‐glycoprotein and rheumatoid factor were reduced. Moreover, in TLR9^−/−^ mice, streptococcal cell wall (SCW)‐induced arthritis was reduced in the T cell‐dependent phase, whereas T cell‐independent serum‐transfer arthritis was not affected. Remarkably, while TLR7 expression did not change during in vitro osteoclastogenesis, TLR9 expression was higher in precursor cells than in mature osteoclasts and partial inhibition of osteoclastogenesis was achieved only by the TLR9 antagonist. These results demonstrate a pivotal role for TLR9 in the T cell‐dependent phases of inflammatory arthritis and additionally suggest some role during osteoclastogenesis. Hence, endogenous DNA seems to be crucially involved in the pathophysiology of inflammatory autoimmune arthritis.

## 1 INTRODUCTION

Rheumatoid arthritis (RA) is the most common and most severe inflammatory joint disease. Apart from cartilage degradation, the predominant clinical feature of RA is bone erosion leading to irreversible destruction of the joints. The onset of RA is characterized by hyperplasia of the synovial lining and infiltration of immune cells such as neutrophils, T cells, B cells, dendritic cells (DC) or macrophages in the synovia of the joint.^1^ In the past years, research has been particularly focused on autoantibodies and to a lesser extent also on autoreactive T cells. In the course of these investigations, the important role of the innate immune system in eliciting the autoimmune responses involved in RA has become increasingly evident. A family of pattern recognition receptors, the Toll‐like receptors (TLRs), is known to bridge the gap between innate and adaptive immunity. TLRs are highly expressed by antigen‐presenting cells (APCs) and recognize distinct ligands, predominantly components of bacteria, viruses or fungi, which are referred to as microbe‐associated molecular patterns (MAMPs). Endogenous TLR ligands, which are expressed or released under stress, are called danger‐associated molecular patterns (DAMPs), such as heat shock proteins, some nuclear proteins and nucleic acids.^2^, ^3^, ^4^, ^5^ To date, 13 TLRs are known in mammals, of which TLR1 to 11 are conserved between humans and mice. Cell surface‐expressed TLRs are the TLRs 1, 2, 4, 5, 6 and 10, which are activated by, for example lipopolysaccharides (LPS), flagellins or (bacterial) lipoproteins. TLRs 3, 7, 8 and 9 are activated by nucleic acids and are located inside the cell at the surface of endosomes. TLR activation induces downstream signaling via mitogen‐activated protein kinases (MAPK) or transcription factors such as nuclear factor of κB (NFκB) or interferon regulatory factor (IRF), resulting in the expression of inflammatory cytokines and type I interferon.^6^ Besides their central function in modulating immune responses, there is evidence that TLR activation is involved in the processes causing the loss of tolerance in RA. A role for TLRs in the pathogenesis of RA has been suggested because these receptors, especially TLR2, 3, 4 and 7, are highly expressed in the synovial tissue of RA patients.^7^, ^8^ Intra‐articular injection of streptococcal cell wall (SCW) lysates containing components of *Streptococcus pyogenes* that activate TLR2 results in acute T cell‐independent joint inflammation^9^ while repeated intra‐articular exposure to SCW fragments results in TLR4 activation and the development of a chronic destructive arthritis depending on T cells.^10^, ^11^ Furthermore, endogenous RNA released from necrotic cells in the synovial fluid of RA patients has been shown to activate synovial fibroblasts through TLR3.^4^ In addition, involvement of TLR3 and double‐stranded (ds) RNA in pristane‐induced arthritis (PIA) has been suggested.^12^ However, the involvement of TLRs activated by single‐stranded (ss) nucleic acids in the inflammatory processes of RA is not fully understood. It was recently shown that TLR7 expression is elevated in synovial fluid monocytes of RA patients and correlates with increased tumour necrosis factor (TNF)‐α levels.^13^ In addition, reduction of collagen induced arthritis (CIA) in TLR7^−/−^ mice points towards the involvement of the RNA‐binding TLR7 in disease maintenance.^14^ Other studies suggest a role for TLR9, which senses unmethylated ssCpG DNA.^15^ It has been demonstrated that unmethylated CpG motifs from bacterial DNA can induce arthritis by activating macrophages and their cytokine production indicating a pathogenic role for bacterial DNA in septic arthritis.^16^ Consequently, inhibition of TLR9 by a suppressive ODN in CpG‐induced arthritis was shown to improve the clinical outcome.^17^ In contrast, activation of TLR9 in mice with K/BxN serum transfer arthritis led to a disease reduction.^18^ Furthermore, involvement of nucleic acid recognizing TLRs in the pathogenesis of autoimmune arthritis has been reported in PIA. In this model, arthritis could be transferred into *naïve* recipients with T cells that had been re‐activated in vitro by the nucleic acid binding protein hnRNP‐A2/B1 or ligands (nucleic acids) of TLR3, TLR7 or TLR9.^19^ Interestingly, disease transfer was inhibited by chloroquine or pre‐treatment of cells with nucleases.

To dissect the role of TLR9 in the pathogenesis of RA, we induced arthritis in TLR9^−/−^ mice and blocked TLR9 activation in PIA by applying suppressive ODNs which have previously been shown to block TLR9 on murine and human cells in vitro.^20^, ^21^ The data obtained demonstrate involvement of TLR9 in the T cell‐dependent phases of erosive arthritis, suggesting a pathogenic role of endogenous DNA and TLR9 in the initiation of arthritogenic autoimmune responses.

## 2 MATERIALS AND METHODS

### 2.1 Toll‐like receptor antagonists and agonists

Antagonists for TLR7 (IRS 661), TLR9 (IRS 869), the TLR9 agonist (1018 ISS) and a control ODN were purchased from Eurofins Genomics (Eurofins Genomics, Ebersberg, Germany). The sequences were previously published.^20^, ^21^ Resiquimod (R‐848) served as TLR7 agonist (Sigma‐Aldrich, St. Louis, USA). For in vitro analysis by flow cytometry, IRS 869 and the control ODN had a fluorescein modification at the 5′‐end (Eurofins Genomics).

### 2.2 Animals

DA.1F rats were bred under conventional conditions at the Institute for Biomedical Research, Medical University of Vienna, Austria. C57BL/6 mice were obtained from Jackson Laboratory. In some experiments, TLR9^−/−^ mice (on a C57BL/6 background) and their respective littermate control mice (TLR9^+/+^) were used.^15^ All experiments were carried out in accordance to EU Directive 2010/63/EU for animal experiments and were approved by the respective local ethics committees.

### 2.3 Pristane‐induced arthritis (PIA)

Arthritis was induced in DA.1F rats by subcutaneous (s.c.) injection of 100 μL pristane (Sigma‐Aldrich) at the tail base. Antagonist (250 μg), control ODN (250 μg) or vehicle was injected s.c. 2 times weekly, and animals were treated twice before disease induction. Inhibitors were diluted in saline to a final volume of 100 μL. Phosphate‐buffered saline (PBS) served as vehicle control. Rats were evaluated for arthritis symptoms every other day starting at day 10 after disease induction using a semi‐quantitative scoring system.^22^ Changes in body weight served as an additional measure of disease severity.^22^

### 2.4 K/BxN serum‐transfer arthritis

C57BL/6 mice were injected with 150 μL K/BxN serum intraperitoneally (i.p.) on day 1 and on day 3. Clinical scoring was started after the first serum application using a semi‐quantitative scoring system. Arthritic limbs were scored from 0 to 3 with a maximum score of 3 per limb and 12 per animal. Grip strength was evaluated as an additional parameter of arthritis. The animals were sacrificed on day 10 after serum application.

### 2.5 Induction of chronic SCW arthritis

Cell wall fragments from *Streptococcus pyogenes* T12 were prepared as described.^23^ Unilateral chronic arthritis was induced by 4 weekly intra‐articular injections of 25 μg SCW in 6 μL PBS into the right knee joint at days 0, 7, 14 and 21. The left joint was injected with saline as control.

### 2.6 Quantification of joint swelling in SCW arthritis

Longitudinal joint swelling at different phases of SCW arthritis was measured by accumulation of ^99m^Technetium (^99m^Tc) in the inflamed joint on days 1 and 2 (acute phase), days 8, 15 and 22 (reactivation days after each intra‐articular injection), and days 23 and 28 as the chronic T cell‐dependent phase.^11^ Swelling was calculated as ratio of gamma counts in the right (inflamed) over the left (internal control) knee joint. Values higher that 1.1 were considered inflammation.

### 2.7 Histological evaluation of joint inflammation and local bone destruction

Hind paws were prepared, fixed in formalin for 24 hours and bones decalcified. Serial paraffin sections were stained with haematoxylin/eosin (H&E), toluidine blue (TB) or were stained for tartrate‐resistant acid phosphatase (TRAP) expression using leucocyte acid phosphatase kit (Sigma‐Aldrich). Histomorphometrical analysis was accomplished using the Osteomeasure software (Osteomeasure 7, Osteometrics, Georgia, USA).

### 2.8 In vitro osteoclast formation assay

Murine bone marrow cells were obtained from femora and tibiae by cutting the end of the bones and flushing them with α‐MEM (Gibco, Thermo Fisher Scientific, Waltham, USA). The cells were cultured for 3 days in α‐MEM supplemented with 10% FCS, 1% penicillin‐streptomycin (all from Gibco) and 100 ng/mL M‐CSF (R&D Systems, Minneapolis, USA). After 3 days, adherent cells were seeded into 96‐well plates (1 × 10^6^ cells/mL) and incubated with 30 ng/mL M‐CSF and 50 ng/mL RANKL (R&D Systems) with or without TLR agonists or antagonists. Osteoclasts were identified by TRAP‐staining (ie, TRAP‐positive cells with ≥3 nuclei), and mean numbers of osteoclasts were calculated out of three wells.

### 2.9 Isolation of cells for FACS analysis and cell culture

Splenocytes from rat or mouse spleens were isolated by passing them through a nylon mesh and cultured in DMEM (Gibco) supplemented with 10% FBS (Sigma‐Aldrich) and penicillin/streptomycin (Gibco). Cells were seeded into 48‐well plates and cultured in the presence of the TLR9 agonist. The TLR9 antagonist, the TLR7 antagonist or the control ODN were added in increasing concentrations. Splenocytes grown in medium alone served as negative control, cells grown in the presence of the agonist only served as positive control. Activation of cells was determined by measuring Interleukin (IL)‐6 in supernatants after 48 hours.

For analysis of TLR antagonist uptake, cells were seeded into 24‐well plates and incubated overnight (o/n) with 1000 nmol/L of fluorescein‐labelled TLR9 antagonist or control ODN. After o/n incubation the medium was removed, and the cells were incubated for up to 72 hours without the antagonists. Uptake of oligodeoxynucleotides was analyzed by flow cytometry.

### 2.10 Real‐time quantitative polymerase chain reaction (RT‐qPCR)

Total RNA from cells was isolated using the RNeasy mini kit (Qiagen, Hilden, Germany). RNA was reverse transcribed into cDNA using the Omniscript RT kit (Qiagen). RT‐qPCR was performed using LightCycler technology (Roche, Germany) and the Fast Start SYBR Green I kit. The expression of the target molecule was normalized to GAPDH. The primer sets used (all from Sigma‐Aldrich) are listed in Table S1.

### 2.11 RT‐qPCR from tissue extracts

Rat organs (dLN and spleens) were mechanically homogenized in TRIfast (Peqlab), followed by isolation of total RNA according to the manufacturer's instructions. mRNA was reverse transcribed into cDNA using the Omniscript RT kit (Qiagen).

### 2.12 Detection of cytokines

Serum samples were obtained from anaesthetized mice and rats by puncture of the heart. The acute phase marker α‐1‐acid‐glycoprotein (AGP) and rat IL‐6 were measured by ELISA obtained from Life Diagnostics (West Chester, PA, USA) and BD Pharmingen, respectively. ELISAs for mouse IL‐6, TNF‐α and IL‐10 were from Thermo Fisher Scientific.

### 2.13 Detection of rheumatoid factor

Rheumatoid factor (RF) levels in rat sera were quantified by ELISA as published^24^ with minor modifications. ELISA plates (NUNC, Roskilde, Denmark) were coated o/n at 4°C with 10 g/L rabbit IgG (Sigma). Isotype‐specific mouse anti‐rat IgM (Pharmingen, San Diego, CA, USA) or goat anti‐rat IgG antibody (Vector, Burlingame, CA, USA) conjugated to biotin was used for detection, followed by incubation with StreptAvidin‐conjugated HRP (R&D Systems). The relative concentrations of RF were determined using high‐titre reference sera obtained from rats with PIA.

### 2.14 Statistical analysis

GraphPad Prism 6 (GraphPad Software, San Diego, CA, USA) was used for graphs and to calculate mean, standard error of the mean (SEM), and statistical significance. Arthritis and grip strength scores are graphically presented as mean ± SEM. The area under the curve (AUC) was used to describe the time course of arthritis and grip strength scores. Statistics were calculated using a two‐tailed non‐parametric Mann‐Whitney U test. For statistical analysis of in vitro experiments where cells of the same specimen were treated with different compounds (ie, splenocytes and osteoclast assay), the paired two‐tailed t test was used. *P*‐values < .05 were considered to be statistically significant.

## 3 RESULTS

### 3.1 Antagonizing TLR9 reduces the severity of pristane‐induced arthritis

To investigate the role of TLR9 in autoimmune arthritis, we used an antagonist that specifically blocks this TLR. To determine its efficacy, the inhibitor was first tested on rat and mouse splenocytes. The antagonist blocked TLR9‐induced secretion of IL‐6 dose dependently (Figure S1A‐C). To demonstrate uptake and internalization of the antagonist, splenocytes were incubated with the fluorescein‐labelled TLR9 antagonist. The data showed that the antagonist was readily taken up by 50%‐60% of the cells and that the signal was stable for at least 72 hours (Figure S1D). Furthermore and in line with this observation, the TLR9 inhibitor significantly reduced secretion of IL‐6, TNF‐α and IL‐10 by mouse splenocytes (Figure S1E‐G).

**Figure 1:**
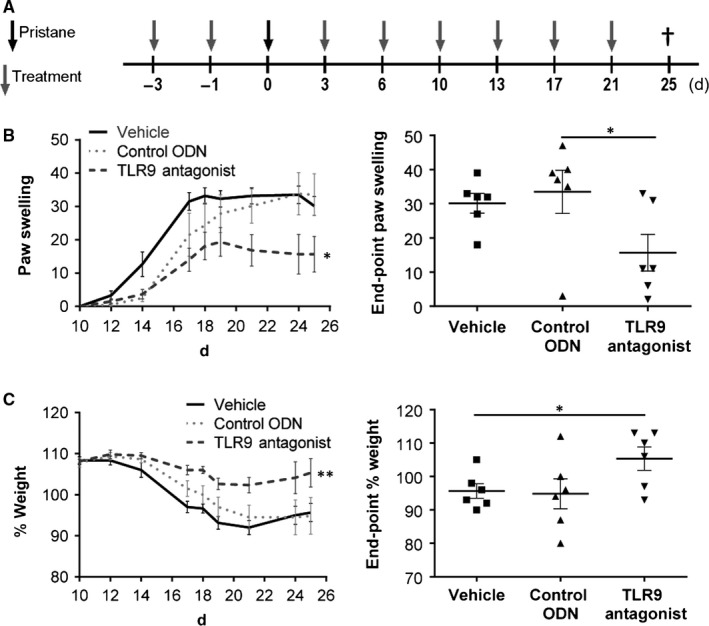
Treatment with the TLR9 antagonist IRS 869 reduces severity of PIA. A, DA.1F rats were prophylactically treated with the TLR9 antagonist, the control ODN or vehicle (phosphate‐buffered saline, PBS) according to the treatment scheme. B, Paw swelling was assessed throughout the experiment starting at day 10, and end‐point paw swelling was calculated for all groups at day 25. C, Weight changes were measured and calculated relative to the starting weight (day 0); end‐point weight change was calculated for all groups at day 25 after disease induction. Graphs show mean ± SEM with *P < .05 (n = 6 animals/group)

To investigate the influence of TLR9 on the development of PIA, we injected the TLR9 inhibitor into DA.1F rats 2 days before inducing arthritis. The rats were then treated twice a week with the antagonist, using a non‐specific ODN and saline as controls (Figure 1A). Development of PIA was assessed until the rats were sacrificed on day 25 post‐pristane injection. Rats treated with the TLR9 antagonist developed milder clinical signs of arthritis and showed significantly reduced paw swelling on day 25 compared with the control groups (Figure 1B). Furthermore, loss of body weight was delayed and much less pronounced in the group of the TLR9 antagonized rats in comparison with the control groups, resulting in a significantly higher end point body weight in the treatment group (Figure 1C).

**Figure 2:**
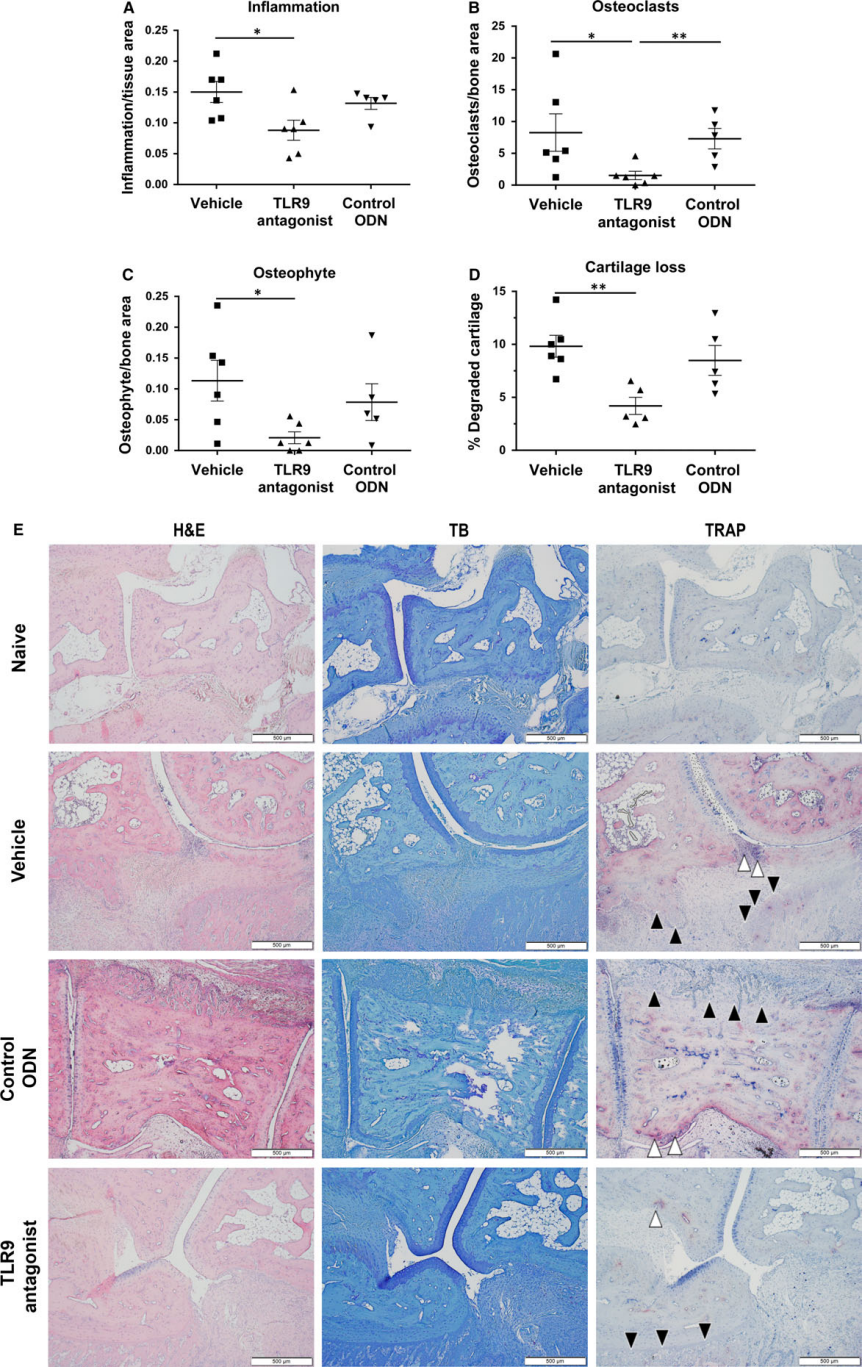
Antagonizing TLR9 leads to reduced inflammation, cartilage degradation and osteoclast numbers in arthritic paws of rats with PIA. Formalin‐fixed paws were stained with H&E, toluidine blue and for TRAP. A, Inflammation, B, number of osteoclasts, C, osteophyte area and D, cartilage loss was analyzed. Quantification was performed as described. Animals treated with the TLR9 antagonist show a reduction in all analyzed parameters compared to animals treated with vehicle or control ODN. E, A representative picture is shown for each treatment group and a naïve animal, where white arrows indicate osteoclasts and black arrows are indicative of osteophyte areas. Graphs represent mean ± SEM with *P < .05 and **P < .01 (n = 5‐6 animals/group)

Clinical observations were confirmed by histomorphometric quantification of paraffin‐embedded sections from tarsal joints (Figure 2). Analysis of invading inflammatory cells into the joint revealed a significant decrease in the inflammation area in TLR9 antagonized rats compared to the control group (Figure 2A,E). In line with this, the number of bone resorbing osteoclasts was diminished in the TLR9 antagonist group (Figure 2B,E). In addition, the osteophyte area was reduced in TLR9 antagonized rats as compared to the control groups (Figure 2C). Analysis of toluidine blue‐stained cartilage sections revealed a significant reduction in cartilage degradation in joints of TLR9 antagonized animals (Figure 2D,E).

**Figure 3:**
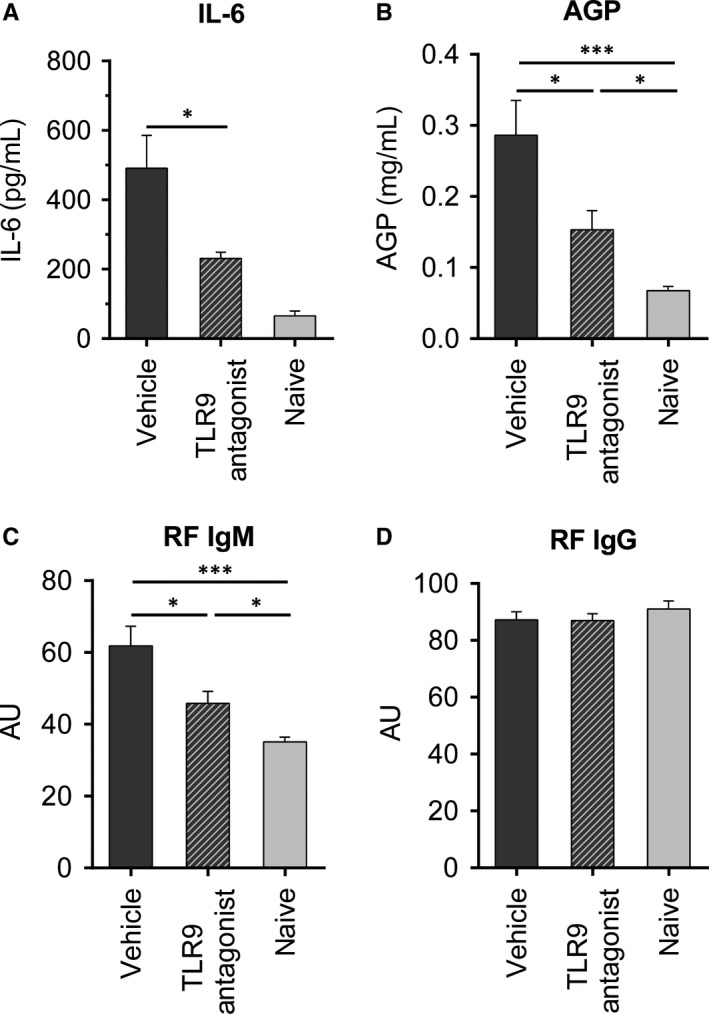
Serum levels of IL‐6, AGP and RF are reduced in rats treated with the TLR9 antagonist. The TLR9 antagonist or vehicle (phosphate‐buffered saline, PBS) was prophylactically administered to DA.1F rats as described. Serum samples were obtained from anaesthetized rats by puncture of the heart on the day of termination of the experiment. Levels of A, IL‐6, B, alpha‐1‐acid glycoprotein (AGP), C, RF‐IgM and D, RF‐IgG were determined by ELISA. Graphs show mean ± SEM with *P < .05 and ***P < .005 (n = 6 for A; n = 8‐9 for B, C and D with data pooled from two individual experiments)

Reduction in arthritis was accompanied by reduced levels of IL‐6 (Figure 3A) and the inflammatory marker alpha‐1‐acid glycoprotein in sera of animals treated with the TLR9 antagonist (Figure 3B). The occurrence of IgM‐RF in PIA has previously been shown to correlate with arthritis development.^24^ In line with these observations, levels of IgM‐RF, but not IgG‐RF were significantly reduced in animals that had received the TLR9 antagonist (Figure 3C,D).

### 3.2 Therapeutically administered TLR9 antagonist has no effect on the progression of PIA

**Figure 4:**
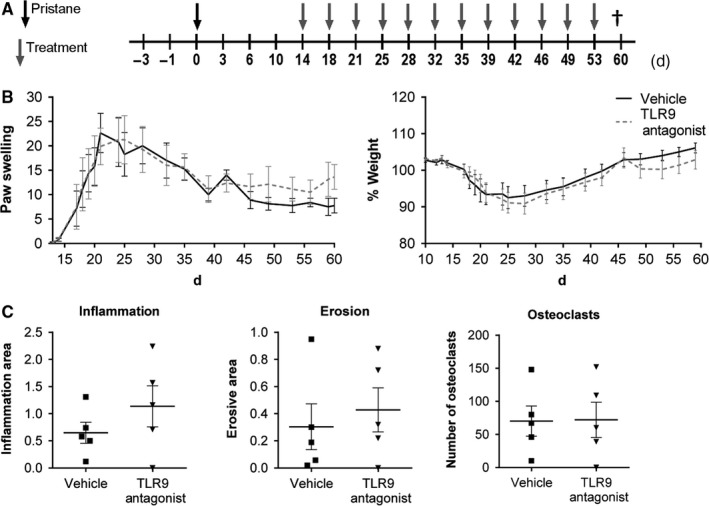
Therapeutic treatment with the TLR9 antagonist does not lead to improvement of PIA. A, Animals were therapeutically treated with the TLR9 antagonist, starting at disease onset (day 14). B, Paw swelling and weight changes were measured throughout the course of PIA. In addition, formalin‐fixed paws were analyzed and C, inflammation, erosion and number of osteoclasts was determined. Results are shown as mean ± SEM with n = 5‐8 animals/group

Next we investigated whether the TLR9 inhibitor could affect arthritis when administered therapeutically after disease induction. At the onset of arthritis (day 14), the rats were treated twice a week with the antagonist or vehicle until they were sacrificed on day 60 (Figure 4A). However, in contrast to prophylactic application, therapeutically administered TLR9 inhibitor did not influence the clinical severity of arthritis (Figure 4B). In accordance with the clinical observation, inflammation, bone erosion and number of osteoclasts in the tarsal joints were not influenced (Figure 4C).

### 3.3 Expression of nucleic acid sensing TLRs in lymphoid organs

Pristane is known to quickly accumulate in draining LNs (dLN) following injection,^25^ leading to an increased number of apoptotic cells in the dLNs within 1‐3 days.^26^ To further analyse the involvement of TLR9 in the development of PIA, we isolated mRNA from spleen and the inguinal (ie*,* draining) LNs. In addition to TLR9, expression of TLR3 (dsRNA binding) and TLR7 (ssRNA binding) was assessed by RT‐qPCR. There were no differences of TLR3, 7 and 9 levels in dLNs of rats treated with the TLR9 antagonist, control ODN or vehicle. However, TLR9 expression was significantly decreased in all three groups in comparison with the naïve control animals (Figure S2A‐C). In contrast to LNs, no differences in TLR expression could be detected between spleens of arthritic and naïve rats (Figure S2D‐F).

### 3.4 Lack of a functional TLR9 does not affect K/BxN serum‐transfer arthritis

**Figure 5:**
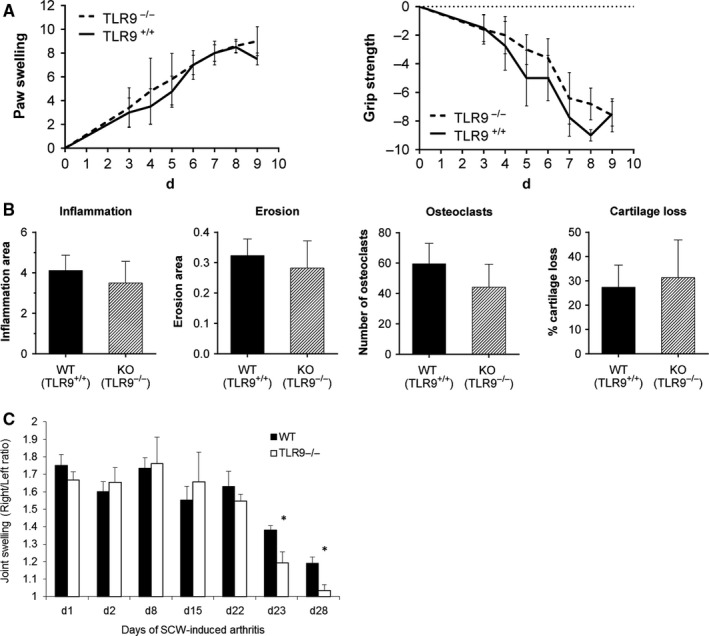
Lack of TLR9 leads to a reduced T cell‐dependent phase of chronic SCW‐arthritis. A and B, K/BxN serum‐transfer arthritis was induced in TLR9^−/−^ and control wild‐type mice. A, Clinical observation of wild‐type (WT, TLR9^+/+^; n = 5) or TLR9 knock‐out (KO, TLR9^−/−^; n = 6) C57BL/6 mice with K/BxN serum‐transfer arthritis and B, histological quantification of the tarsal joints of these mice revealed no differences in inflammation, bone erosion, number of osteoclasts (ie, TRAP‐positive cells) or cartilage loss in TLR9^−/−^ animals as compared to wild‐type mice. C, SCW arthritis was induced in TLR9^−/−^ (n = 6) and control wild‐type (n = 6) mice by repeated intra‐articular injections of 25 μg SCW into the right knee joint at days 0, 7, 14 and 21, and injection of saline as internal control into the left joint. Joint swelling was determined by measuring ^99m^Tc uptake by the right (inflamed) joint compared to the left (control) joint at several time points including the acute (d1 and d2), reactivation (d8, d15 and d22) and the chronic (d23 and d28) phase of arthritis. Right/Left ratio of the radioactive ^99m^Tc signal is depicted on the *Y*‐axis. Results are presented as mean ± SEM with *P < .05 by Mann‐Whitney U test versus WT mice

To further dissect the involvement of TLR9 in T cell‐dependent versus T cell‐independent phases of inflammatory arthritis, we investigated disease development and progression in the K/BxN serum‐transfer model which is independent of T cells and characterized by a fast developing monophasic destructive arthritis in all tarsal joints.^27^ C57BL/6 mice lacking a functional TLR9 (TLR9^−/−^) and their littermate wild‐type controls (TLR9^+/+^) were injected with K/BxN serum to induce arthritis. TLR9 deficiency did not result in any alterations in paw swelling or grip strength (Figure 5A). In accordance with the clinical observation, the inflamed area, bone erosions, number of osteoclasts and cartilage destruction of the tarsal joints were not influenced by the lack of TLR9 (Figure 5B). Thus, TLR9 did not seem to have an influence on incidence and severity of serum‐transfer arthritis.

### 3.5 TLR9 is involved in the T cell‐dependent phase of chronic SCW arthritis

To confirm the differential roles of TLR9 in T cell‐dependent and T cell‐independent arthritic processes, we next investigated the involvement of TLR9 in the course of repeated SCW arthritis, which is characterized by a T cell‐independent initial phase after the first intra‐articular SCW injection, followed by a T cell‐dependent chronic phase after the fourth SCW reactivation.^11^ We induced SCW arthritis in TLR9^−/−^ and control wild‐type mice, and quantified joint inflammation. Joint swelling was not affected by TLR9 deficiency in the acute phase of SCW arthritis, that is days 1 and 2 after the first SCW injection (Figure 5C). Furthermore, the intermediate reactivation phase of arthritis post each intra‐articular injection (days 8, 15 and 22) was similar between wild‐type and TLR9^−/−^ mice. However, the T cell‐dependent chronic phase of SCW arthritis was significantly suppressed in TLR9^−/−^ mice as demonstrated by reduced joint swelling on days 23 and 28 (Figure 5C).

### 3.6 TLR9 is differentially expressed in the course of in vitro osteoclastogenesis

As the number of osteoclasts was substantially reduced in TLR9 antagonized rats, we became interested to analyze the impact of TLR9 on in vitro osteoclast formation using bone marrow cells from wild‐type mice, which were cultivated in the presence or absence of TLR9 and TLR7 antagonists or agonists, or a control ODN.

**Figure 6:**
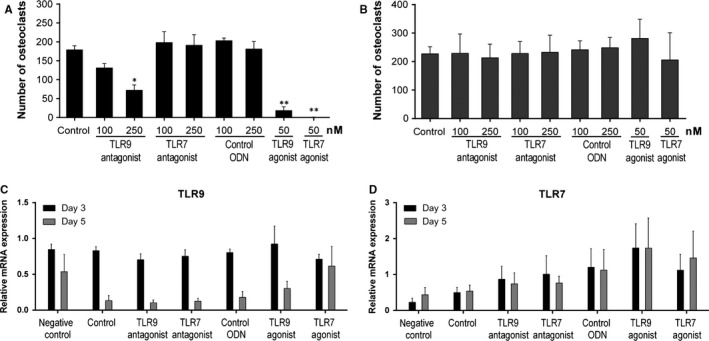
Involvement of TLR9 and TLR7 in RANKL‐induced osteoclastogenesis. Bone marrow‐derived murine cells from wild‐type mice were expanded with M‐CSF and subsequently incubated with M‐CSF and RANKL (day 0) to induce differentiation into osteoclasts. Antagonists of TLR9, TLR7, a control sequence (control ODN), or agonists of TLR9 (CpG) and TLR7 (R‐848) were added A, either together with M‐CSF and RANKL or B, 3 days after treatment with M‐CSF and RANKL. Osteoclasts (ie, multinucleated TRAP‐positive cells) were identified by TRAP staining. C and D, To analyze expression of TLR9 and TLR7 during osteoclastogenesis, cells were grown in the presence of TLR antagonists (250 nmol/L) or agonists (50 nmol/L) or a control ODN (250 nmol/L). Cells grown in the presence of M‐CSF only served as negative control. Total RNA was isolated at different time points (ie, day 0, day 3 and day 5) during osteoclast formation in the presence of RANKL. TLR expression was analyzed by RT‐qPCR and calculated relative to expression on day 0. Bars are represented as mean ± SEM of three independent experiments with *P < .05 and **P < .01 (compared to control)

When the cells were exposed to TLR9 and TLR7 antagonists or agonists, the formation of osteoclasts was reduced by approximately 40% by the TLR9 but not the TLR7 antagonist or the control ODN (Figure 6A). Furthermore, in accordance with previously published observations,^28^, ^29^ both agonists completely blocked osteoclast formation (Figure 6A). As the TLR9 agonist CpG has been shown to exert different effects during in vitro osteoclastogenesis depending on the time‐point of the exposure to CpG, leading either to enhanced or reduced osteoclast formation,^29^ we sought to explore whether agonists and antagonists of TLR9 and TLR7 would still exert a suppressive effect when cells had been pre‐incubated with RANKL. Indeed, when cells were stimulated with RANKL 3 days prior to addition of the agonists or antagonists, the inhibitory effect observed for the TLR9 agonist as well as the TLR9 antagonist was completely abolished as was the inhibitory effect of the TLR7 agonist (Figure 6B).

In addition, we analyzed the expression of TLR9 and TLR7 by RT‐qPCR at different time points during in vitro osteoclastogenesis. In osteoclast precursor cells, TLR9 expression was high on day 3 but decreased to almost background levels after 5 days (Figure 6C). TLR9 expression was not influenced by antagonists but on day 5 appeared to be slightly, although not significantly, increased by the agonists, particularly by the TLR7 agonist (R‐848). Expression of TLR7 did not change between day 3 and day 5, but increased by ~2‐fold upon culture with the TLR7 or the TLR9 agonist, but this increase did not reach statistical significance (Figure 6D). Altogether, these data suggest a dynamic regulation of TLR9 expression and a phase‐dependent role for this receptor during osteoclastogenesis.

## 4 DISCUSSION

It is now widely accepted that RA is initiated by autoreactive T cells leading to the production of pathogenic autoantibodies such as RF or antibodies to citrullinated proteins (ACPA). However, the triggering events are still largely unknown and the autoantigens activating arthritogenic T cells have not yet been identified. Nevertheless, based on data obtained in patients with RA and animal models, it is conceivable that TLRs and their endogenous ligands may play a pivotal role in the pathogenesis of RA. Apart from overexpression of various TLRs in the synovium of RA patients,^7^, ^8^ this has been particularly demonstrated for TLR7 in CIA,^14^ for both TLR2 and TLR4 in SCW‐induced arthritis^9^, ^10^, ^30^, ^31^ and also for TLR3 in PIA.^12^ In previous investigations, we showed that PIA can be transferred into naïve recipient rats by CD4^+^ T cells after in vitro re‐stimulation with autologous APCs.^19^ Activation by nucleic acids or nucleic acid‐containing proteins (eg, hnRNP‐A2/B1) was required for the APCs to regain their stimulatory capacity, suggesting a crucial involvement of nucleic acid‐binding TLRs and their endogenous ligands in initiation of PIA.^19^ In accordance with these findings, we could now demonstrate that blocking TLR9 activation led to a significant amelioration of arthritis when the TLR9 antagonist was applied before disease induction. Reduction in clinical severity was supported by histological examination and also by serological findings, revealing decreased levels of the pro‐inflammatory cytokine IL‐6, the acute phase protein AGP and the autoantibody IgM‐RF in sera of animals treated with the TLR9 antagonist. Reduced serum levels of IL‐6, AGP and IgM‐RF may explain the early treatment effect as this could affect the T cell activation in the first priming phase. It has been clearly shown that pristane activates APCs priming autoreactive T cells, which induces a chronic arthritis development.^32^ Thus, we assume that the major effect of TLR9 inhibition is suppression of cytokine production and reduced activation of APCs, leading to reduction in inflammation and partial suppression of arthritogenic T cell responses. This is also supported by in vitro data showing substantially reduced secretion of IL‐6, TNF‐α and IL‐10 by splenocytes treated with the TLR9 antagonist. However, when the antagonist was administered therapeutically after the onset of arthritis, no protective effects were observed, indicating that PIA becomes independent of TLR9 signaling and T cells once the disease process has been set in motion.

In line with these observations, SCW‐induced arthritis in TLR9^−/−^ mice was reduced only in the T cell‐dependent phase. In contrast to PIA, the acute phase of SCW‐induced arthritis is TLR2 dependent but shifts towards a TLR4 dependency in the chronic phase, in which TLR2 is no longer relevant. This phase is driven by T cells and completely abrogated in RAG2‐deficient mice.^10^, ^11^, ^30^ In fact, chronic SCW arthritis is also accompanied by bone destruction and enhanced expression of RANK, RANKL and cathepsin K protein in cortical bone, features that are also characteristic of RA. We have previously observed that in the chronic SCW‐induced arthritis model, the expression of TLR9 mRNA is up‐regulated after each SCW injection and the kinetics of TLR9 expression follows the arthritis reactivation pattern, suggesting a potential relevance for TLR9 also in this model.^33^ The data obtained in TLR9^−/−^ mice now clearly demonstrate that TLR9 is indeed involved in this T cell‐dependent phase of chronic SCW‐arthritis, highlighting the relevance of TLR9 in T cell‐mediated arthritic processes, and thus suggesting a potential role for endogenous TLR9 ligands also in SCW‐induced arthritis.

In contrast to SCW‐arthritis, TLR9 deficiency had no influence on K/BxN serum‐transfer arthritis. This model does not involve T cells but rather reflects the effector phase of autoimmune arthritis^27^ and allows for studying the impact of TLR9 on innate immune cells.^34^, ^35^, ^36^ This observation is therefore in full agreement with the ineffectiveness of TLR9 inhibitory treatment when started after the onset of PIA.

The most remarkable histological finding of prophylactic TLR9 inhibition in PIA was the almost complete absence of osteoclasts and bone erosion. It is known that TLR9 stimulation influences the outcome of osteoclastogenesis in a time‐dependent manner, leading to a profound reduction in osteoclastogenesis.^29^ To further investigate this process, we performed osteoclast assays with bone marrow cells that had or had not been incubated with RANKL before adding TLR antagonists or agonists. Indeed, partial inhibition of osteoclastogenesis was achieved with the TLR9 antagonist, whereas the TLR7 antagonist was ineffective. However, this effect was only observed in unprimed, that is RANKL naive cells. It is conceivable that blocking of MAPK and NF‐κB signaling by the TLR9 antagonist led to partial suppression of osteoclast formation, because both pathways are involved in RANKL‐induced osteoclast differentiation. Of note, the previously described suppression of osteoclastogenesis by TLR9 activation was shown to be caused by dephosphorylation of ERK.^37^

Analysis of TLR expression during RANKL‐induced osteoclastogenesis revealed different expression patterns. While expression of TLR7 did not change much, expression of TLR9 was high at the beginning of osteoclastogenesis but very low in mature osteoclasts, indicating a strong down‐regulation of TLR9 by RANKL while neither the TLR antagonist nor the agonist had significant influence on the expression of TLR9. This suggests a modulatory role of TLR9 during osteoclastogenesis and a temporal regulation of TLR9 expression during this process which may explain why TLR9 manipulation (stimulation or inhibition) is ineffective in mature osteoclasts. From all these findings, we assume that TLR9 activation by endogenous ligands plays an important (though not essential) role during initiation of osteoclastogenesis while activation by exogenous ligands (eg, during infection) seems to activate a suppressive mechanism to avoid bone degradation in the course of a massive inflammatory response. However, it must be borne in mind that all these data were obtained in cell culture systems which only partially reflect the much more complex in vivo situation and that TLR9 inhibition did not show any significant effects on bone erosion or osteoclastogenesis (as estimated from the number of TRAP‐positive cells) in serum‐transfer arthritis or in established PIA. Although the data obtained in vitro indicate involvement of TLR9 in osteoclastogenesis, its role in vivo is less obvious and requires further investigations.

How can all these findings be reconciled with human RA? In contrast to systemic lupus erythematosus, antinuclear antibodies (mostly directed to nucleic acid binding proteins) are infrequently observed in RA and antibodies to dsDNA are virtually absent. The hallmark antibodies of RA are antibodies to citrullinated proteins, but the majority of the targets identified so far (eg, fibrinogen, vimentin or alpha‐enolase) are not associated with nucleic acids. Notable exceptions are histones which are citrullinated in vivo*,* especially during apoptosis and NETosis, and hnRNP‐A2/B1.^38^ Importantly, autoantibodies and autoreactive T cells recognizing hnRNP‐A2/B1 and related proteins have been found in PIA and human RA.^39^, ^40^, ^41^, ^42^ Given that most ACPA‐positive RA patients are reactive with citrullinated histone peptides and/or hnRNP‐A2/B1, autoimmunity to citrullinated isoforms of nucleic acid binding proteins appears to be a common phenomenon in RA.

Thus, we propose a pathogenetic model of erosive autoimmune arthritis in which endogenous nucleic acids that are released from dying cells because of an environmental adjuvant trigger (pristane, cigarette smoke, infection, etc.) play a crucial role in the initiation of arthritogenic autoimmune responses (directed to nucleic acid binding proteins) on a predisposing genetic background, particularly MHC class II, because of their ability to activate endosomal TLRs.

Taken together, our data suggest a strong impact of TLR9 on the initiation of T cell‐dependent arthritis, whereas its role in disease‐progression remains elusive as neither therapeutic application of a TLR9 antagonist nor lack of a functional TLR9 ameliorated PIA or serum‐transfer arthritis, respectively. Concerning a potential application of our findings for the treatment of RA, therapeutic application of TLR9 antagonists might be considered in the very early pre‐clinical phases in persons at high risk for developing RA to prevent (further) generation of harmful autoimmune reactions leading to the inflammatory and deleterious processes in the joint characteristic of RA.

## AUTHOR'S CONTRIBUTIONS

A.F., S. A.‐R., C.B., L. J. and G.S. contributed to the design of the study. A.F., S. A.‐R., C.B., B.N., M.B., A. Y. and E. L. contributed to the acquisition, collection and assembly of data. M. K., C. L., D. D., G. K., R. H. and L. J. contributed to reagents/materials/analysis tools. A.F., S. A.‐R. and G.S. wrote the main manuscript text. All authors contributed in revising the manuscript and approved the final version.

## CONFLICT OF INTEREST STATEMENT

The authors confirm that there are no conflicts of interest.
