# Supplementary material for: The involvement of Toll‐like receptor 9 in the pathogenesis of erosive autoimmune arthritis
Source: J Cell Mol Med. 2018 Jul 11;22(9):4399–409. doi: 10.1111/jcmm.13735 (PMC6111819; doi:10.1111/jcmm.13735)
Supplement: Supplementary file 1 [file JCMM-22-4399-s001.docx]

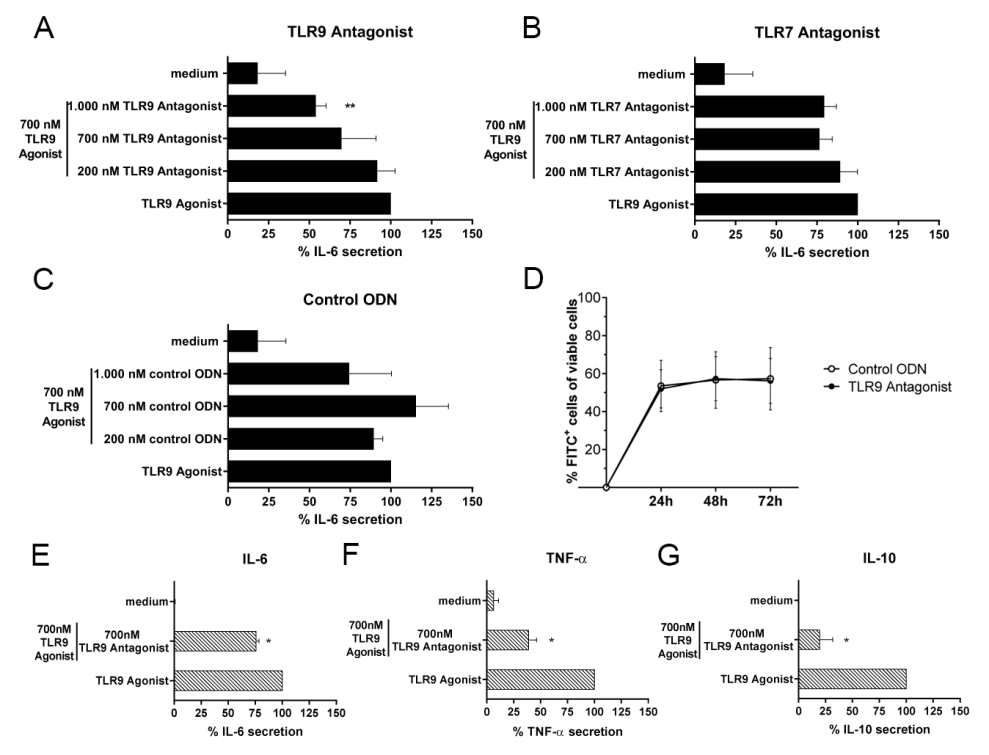


**Figure S1. *In vitro* analysis of the TLR9 antagonist IRS 869.** (A-C) Splenocytes from naïve DA.1F rats were activated via TLR9 using the CpG-containing immunostimulatory sequence 1018 (ISS 1018) and concurrently incubated with increasing doses of the TLR9 antagonist IRS 869, the TLR7 antagonist IRS 661 or an unspecific control oligodeoxynucleotide (ODN). A dose-dependent reduction of IL-6 secretion (measured by ELISA) by the TLR9 antagonist can be seen. (D) In addition, splenocytes were incubated with the fluorescein-labelled TLR9 antagonist or control ODN and their uptake was analyzed by flow cytometry. The ODNs were readily taken up by the majority of splenocytes of which 50 to 60% showed a fluorescent signal. (E-G) In addition, splenocytes isolated from wild-type mice were treated as in (A) and secretion of IL-6, TNF-α and IL-10 was determined by ELISA. Results are shown as mean ± SEM from 3 independent experiments.
