# Supplementary material for: The involvement of Toll‐like receptor 9 in the pathogenesis of erosive autoimmune arthritis
Source: J Cell Mol Med. 2018 Jul 11;22(9):4399–409. doi: 10.1111/jcmm.13735 (PMC6111819; doi:10.1111/jcmm.13735)
Supplement: Supplementary file 2 [file JCMM-22-4399-s002.docx]

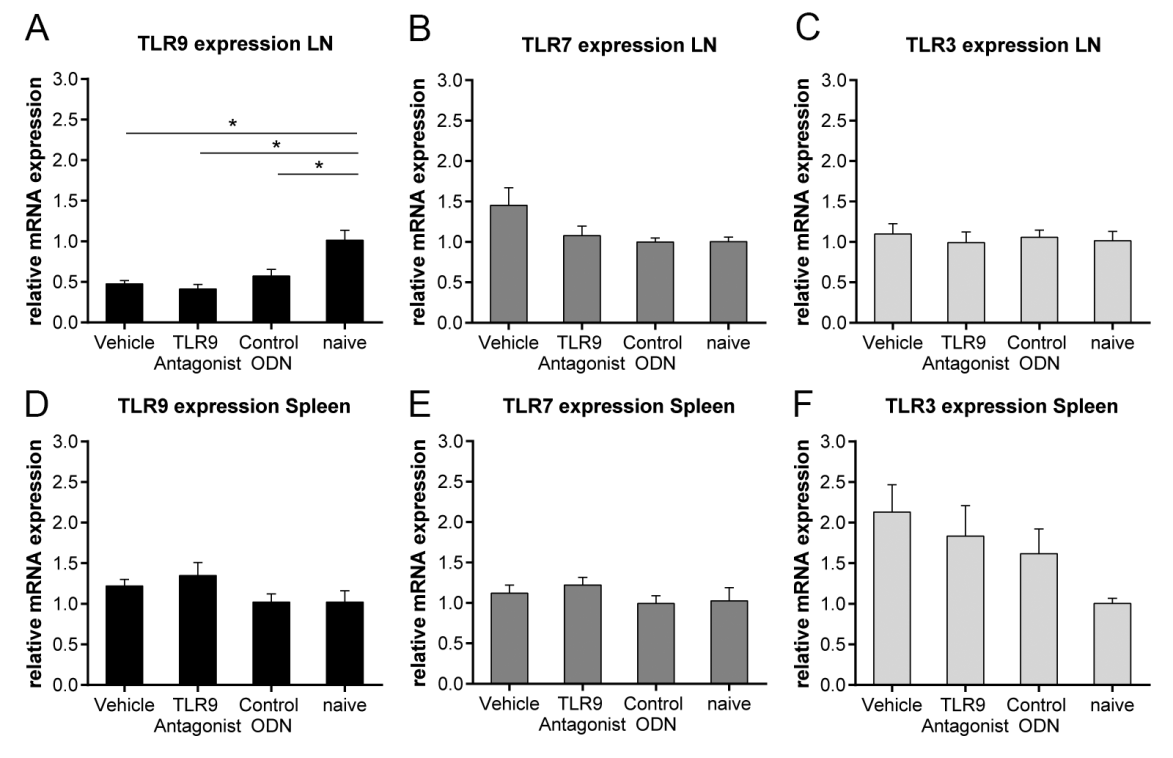


**Figure S2.** **Expression analysis of TLRs in draining lymph nodes and spleens.** Draining LNs and spleens were dissected from rats with PIA 25 days after disease induction for isolation of RNA. Expression of TLR9, 7 and 3 was analyzed by RT-qPCR and calculated relative to expression of naïve animals. (A-C) Expression analysis in LNs reveals significant downregulation of TLR9 expression but stable expression of TLR7 and TLR3 in all 3 treatment groups compared to naïve animals. (D-F) Expression analysis in spleens shows no changes of TLR3, TLR7 and TLR9 expression and no differences between naïve and arthritic animals. Data are shown as mean ± SEM with * = p<0.05 (where the different treatment groups were compared to naïve animals with n=6 animals/group and n=3 for naïve animals).
