# Supplementary material for: The involvement of Toll‐like receptor 9 in the pathogenesis of erosive autoimmune arthritis
Source: J Cell Mol Med. 2018 Jul 11;22(9):4399–409. doi: 10.1111/jcmm.13735 (PMC6111819; doi:10.1111/jcmm.13735)
Supplement: Supplementary file 3 [file JCMM-22-4399-s003.docx]

**Supporting Table 1.** Primer sequences used for RT-qPCR

| **Target Gene** | **Primer Sequence** |
| --- | --- |
| mouse TLR4 | 5’-GCTTGAATCCCTGCATAGAGGTAG-3’ (forward) |
|  | 5’-GTCTCCACAGCCACCAGATT-3´ (reverse) |
| mouse TLR7 | 5’-AAGGCTCTGCGAGTCTCGGTT-3’(forward) |
|  | 5’-GAGAAGGGAGCCAAGGACATCTTTC-3’(reverse) |
| mouse TLR9 | 5’-TGCCGACTGGGTGTATAACG-3’(forward) |
|  | 5’-GTCCTTGCGGTCTTCCAACA-3’(reverse) |
| rat TLR3 | 5’-TGCGATTGGCAAGTTATTCG-3’(forward) |
|  | 5’-GCGGAGGCTGTTGTAGGAAA-3’(reverse) |
| rat TLR4 | 5’-TCCCTGCATAGAGGTACTTC-3’(forward) |
|  | 5’-TCTCCACAGCCACCAGATTC-3’(reverse) |
| rat TLR7 | 5’-TTTCCCAGAGCATACAGCTCAG-3’(forward) |
|  | 5’-CACTCAAGGACAGAACTGCTGC-3’(reverse) |
| rat TLR9 | 5’-CCTGGCACACAATGACATTCA-3’(forward) |
|  | 5’-TAAAGGTCCTCCTCGTCCCA-3’(reverse) |
| GAPDH | 5’-TGGCATTGTGGAAGGGCTCATGA-3’(forward) |
|  | 5’-ATGCCAGTGAGCTTGCCGTTCAG-3’(reverse) |
